# Mechanically activated artificial cell by using microfluidics

**DOI:** 10.1038/srep32912

**Published:** 2016-09-09

**Authors:** Kenneth K. Y. Ho, Lap Man Lee, Allen P. Liu

**Affiliations:** 1 Department of Mechanical Engineering, University of Michigan, Ann Arbor, Michigan, United States of America; 2 Department of Biomedical Engineering, University of Michigan, Ann Arbor, Michigan, United States of America; 3 Cellular and Molecular Biology Program, University of Michigan, Ann Arbor, Michigan, United States of America; 4 Biophysics Program, University of Michigan, Ann Arbor, Michigan, United States of America

## Abstract

All living organisms sense mechanical forces. Engineering mechanosensitive artificial cell through bottom-up *in vitro* reconstitution offers a way to understand how mixtures of macromolecules assemble and organize into a complex system that responds to forces. We use stable double emulsion droplets (aqueous/oil/aqueous) to prototype mechanosensitive artificial cells. In order to demonstrate mechanosensation in artificial cells, we develop a novel microfluidic device that is capable of trapping double emulsions into designated chambers, followed by compression and aspiration in a parallel manner. The microfluidic device is fabricated using multilayer soft lithography technology, and consists of a control layer and a deformable flow channel. Deflections of the PDMS membrane above the main microfluidic flow channels and trapping chamber array are independently regulated pneumatically by two sets of integrated microfluidic valves. We successfully compress and aspirate the double emulsions, which result in transient increase and permanent decrease in oil thickness, respectively. Finally, we demonstrate the influx of calcium ions as a response of our mechanically activated artificial cell through thinning of oil. The development of a microfluidic device to mechanically activate artificial cells creates new opportunities in force-activated synthetic biology.

Engineering artificial cell to mimic cellular functions is an emerging area of synthetic biology. It is a high-risk, high-reward path towards the advancement of both basic and applied science. Recent success in synthesizing minimal self-replicating cells^1^ helps to answer many basic scientific questions about the minimum genome to support life and also opens up new applications, such as novel vaccine development and sustainable bio-fuels. Bottom-up *in vitro* reconstitution is an alternative approach in engineering artificial cells by which cellular functions are reconstituted by component macromolecules to study cellular assembly and organization^2^. As such, bottom-up reconstitution offers a way to peel away cellular complexity through building from cellular components to understand how macromolecules assemble and organize into complex structures or generate emergent behaviors^3^,^4^, and building bio-inspired artificial cells where inherent complexities of living cells are stripped away holds tremendous promise in a broad range of applications^5^,^6^.

Bottom-up synthesis of cell-like artificial cells faces two general challenges – encapsulation and stability. As a chassis of artificial cells, droplet microfluidics with high encapsulation efficiency has been used to aid the synthesis of liposomes by double emulsion template^7^, layer-by-layer assembly^8^,^9^ and microfluidic jetting^10^,^11^. As a promising approach towards building artificial cells, cell-free expression components were encapsulated within a lipid bilayer platform by droplet microfluidics^12^,^13^,^14^. However, building artificial cell systems directly with lipid bilayer has challenged many research groups, mainly due to low stability of artificial cells. This has motivated us to use double emulsion droplets (aqueous/oil/aqueous) as an alternative model system to prototype artificial cells. Indeed, numerous studies have used single or double emulsions or other compartmentalized schemes as artificial cells^15^,^16^.

Current engineered artificial cells have been limited to sensing chemical inputs or physicochemical properties of membrane^6^,^17^,^18^, while the idea of engineering a mechanosensing artificial cell has never been conceived. Mechanical forces are arguably the most primitive signal and mechanosensation is universal to all living organisms^19^. In principle, a mechanosensing artificial cell could be engineered to sense and respond to mechanical forces, such as shear, tensile, or compression forces. To demonstrate the construction of mechanosensing artificial cell, a precisely controlled platform is required to physically apply mechanical forces to artificial cells. Microfluidics provides a platform with precise microenvironment control and it has been utilized in numerous applications in synthetic biology, ranging from automated DNA assembly^20^,^21^, automated genetic engineering^22^, to precise control of bacterial culture^23^. Microfluidic technology and synthetic biology have also been combined together for studying different genetic circuit systems, such as metabolic gene network^24^, synchronizing genetic oscillations^25^,^26^ and molecular clocks^27^. Recently, microfluidics was used to engineer on-chip artificial cells^16^,^28^. The growth of microfluidics in synthetic biology applications is clearly evident. In the context of the present study, microfluidics can provide precise mechanical input to artificial cells. Microfluidics technology has been widely adopted in single cell mechanobiology studies where the effect of different modes of mechanical forces on cellular functions have been investigated^29^,^30^,^31^,^32^,^33^,^34^. Thus, the capability and controllability of microfluidics offer a versatile platform for engineering and studying mechanically activated artificial cells.

Our long-term goal is to engineer artificial cells that respond to mechanical forces. As a first step, we developed a multilayer microfluidic platform to trap and apply mechanical forces to single artificial cells. We used double emulsions generated from droplet microfluidics as a model of artificial cell. Our novel microfluidic device could compress or aspirate on double emulsion droplets to increase or decrease the thickness of oil in the middle phase, respectively. By thinning the oil phase, we demonstrated the influx of calcium ions as a response of our mechanically activated artificial cell. The influx of extracellular calcium ions is one of the most important signaling pathways found in living cells, where calcium ions serve as secondary signaling messengers and exert regulatory effects on different enzymes and proteins. Our study here combining microfluidics and artificial cells opens up new possibilities in the development of force-activated synthetic biology.

## Results and Discussion

### Model of mechanosensing artificial cell

**Figure 1:**
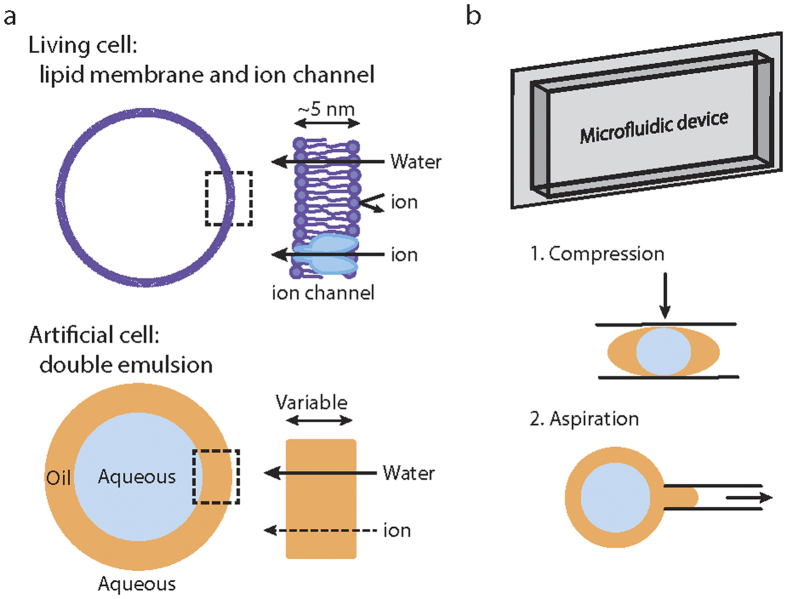
Prototyping mechanosensing artificial cells. **(a)** Lipid bilayer partitions the internal environment of a cell where ions do not cross. Double emulsion can serve as a model of an artificial cell where the oil middle phase can vary in thickness and acts as a semipermeable barrier for ions to pass through. **(b)** A microfluidic device can be utilized to compress or aspirate on double emulsions as a way to alter oil thickness and mechanically activate artificial cells.

In biological systems, selectively permeable membrane is composed of a thin single bilayer of lipid molecules with a thickness of ~5 nm. Membrane proteins such as transporters and ion channels are important in controlling transport of chemicals and ions across the membrane. Mechanosensitive channels that are responsive to membrane tension are found from bacteria (e.g. MscL) to mammalian cells (e.g. Piezo 1)^35^,^36^. Reconstituting mechanosensitive channel activity as a path towards constructing mechanosensitive artificial cell is a challenging, yet promising approach. As an alternative model to lipid bilayer vesicles, double emulsion droplets can be used to prototype artificial cells (Fig. 1a). As water and oil are immiscible with each other, the middle oil phase separates the inner and outer aqueous phases to form double emulsion. The thickness of the oil dictates the transport of hydrophobic solutes via diffusion^37^ or by reverse micelles in the presence of osmotic mismatch^38^. To prototype mechanosensing artificial cells, we investigated the possibility of using compression and aspiration to alter oil thickness in double emulsion as a mechanism to mechanically activate artificial cells (Fig. 1b).

### Microfluidic device overview and design

**Figure 2:**
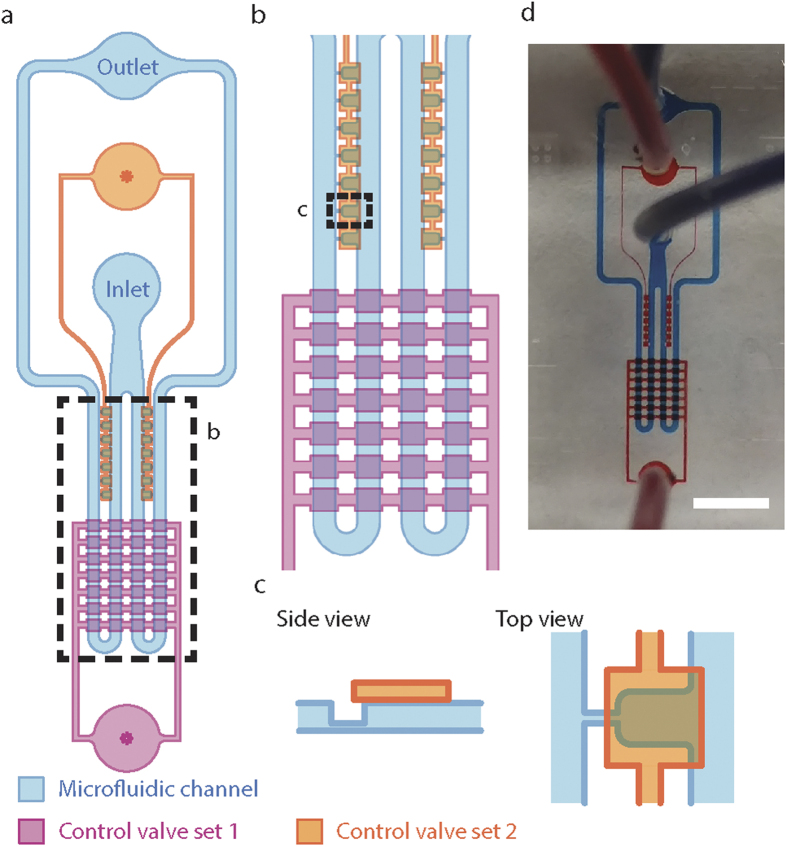
Overview and design of the microfluidic device. **(a)** The design of the microfluidic device is shown. The device has two PDMS layers that consist of a deformable microfluidic channel and two independent control valve sets. **(b)** Close-up view of the control valve sets from the dotted box in (**a**). Control valve set 1 facilitates the trapping of droplets inside the trapping chambers and Control valve set 2 provides in-plane compression of the trapped droplets. **(c)** Top view (right) and side view (left) of a trapping chamber. The microfluidic channel is beneath the control layer and is separated by a thin, deformable PDMS membrane. The pipette structure adjacent to the trapping chamber is located at the bottom of the microfluidic channel. **(d)** A picture of the actual device connected with micro-tubings: blue and red color dyes label the microfluidic channel and control valve sets respectively, scale bar = 3 mm.

To demonstrate the construction of mechanosensing artificial cells, a microfluidic device is designed to trap double emulsions into trapping chambers, and to apply compression and aspiration to the double emulsions in a parallel manner. The device, made out of polydimethylsiloxane (PDMS), has two layers, the flow layer and the control layer (Fig. 2a–d). The flow layer (blue) has a similar design as our previously reported microfluidic pipette array (μFPA) device^34^ where fluid and double emulsions flow from one inlet through the microfluidic channel to one outlet. The microfluidic channel first splits into two channels, each containing 7 trapping chambers. Each trapping chamber is connected to the opposing end of the main microfluidic channel through a small microchannel, akin to a micropipette that can perform aspiration. The flow layer is designed such that the flow resistance of the small microchannel is 20 times larger than that of the main microfluidic channel. Thus, flow is directed primarily through the main microfluidic channel and not through the trapping chambers. A thin PDMS membrane separates the microfluidic channel and the control layers (pink and orange) that serve two different functions and are independently controlled (Fig. 2b). To direct flow to the trapping chambers, a pneumatically controlled valve set located above the microfluidic channel blocks the flow in the main microfluidic channel when the valve set (Control valve set 1, pink) is actuated. A second control valve set (Control valve set 2, orange) directly above the trapping chambers exerts compression to trapped double emulsion when the valve set is actuated. Since the two control valve sets can be independently controlled, trapping is decoupled from compression.

### Membrane deflection and compression by pneumatic control

**Figure 3:**
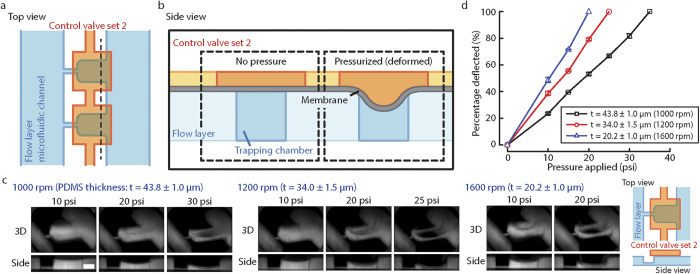
Deflection characterization of PDMS membrane by pressurizing control layer 2. (**a**) Top view: Control valve set 2 is aligned with the trapping chambers in microfluidic channel. (**b**) Side view: Air pressure in Control valve set 2 is regulated by a pressure regulator. At zero pressure, the membrane between Control valve set 2 and microfluidic channel is flat. When Control valve set 2 is pressurized, the membrane deflects. (**c**) Images from confocal microscopy of fluorescent dye perfused in the microfluidic channel. Z-stack images were reconstructed in ImageJ to generate 3D and side view images. The deflections of PDMS membrane with different spin-coating speed under different applied pressure are shown, scale bar = 50 μm. (**d**) The percent deflection of the PDMS membrane as a function of different PDMS spin-coating speed and applied pressure (n = 4). The PDMS membrane thickness *t* (mean ± SEM) was measured for each spin-coating speed.

One of the features that the microfluidic device has is to compress trapped double emulsions in the trapping chambers. Control valve set 2 features rectangular patterns above each trapping chamber. When Control valve set 2 is pressurized, the PDMS membrane above the trapping chambers deflects and compresses the double emulsions trapped in the trapping chambers. The rectangular patterns of Control valve set 2 needs to be aligned with the trapping chambers of the microfluidic channel (Fig. 3a) such that deflection of the PDMS membrane is directly over the trapping chambers. Without actuation, the membrane remains flat. When air pressure in Control valve set 2 increases and is higher than the liquid pressure in the microfluidic channel, the PDMS membrane deflects towards the flow channel (Fig. 3b). The deflection of the membrane is dependent on the material property of the PDMS membrane, the thickness of the membrane and the applied pressure in Control valve set 2. To fabricate PDMS membrane with a higher elasticity for better deflection compared to PDMS with 10:1 (base:curing agent) mixing ratio^39^, we chose a higher mixing ratio of 20:1 to make the PDMS membrane. By varying the PDMS spin-coating speed on the flow layer silicon mold (1000–1600 rpm), we can generate PDMS membrane with thicknesses ranging from 20 μm to 44 μm. To examine the deflections of membrane with different thicknesses as a function of different applied pressures, we labeled the microfluidic channel volume with rhodamine succinimidyl dye since we cannot directly label the PDMS membrane. When Control valve set 2 is pressurized, the membrane deflects and displaces the fluid in the microfluidic channel so that we can indirectly visualize membrane deflection. The reconstructed 3D and side view images of the compressed trapping chambers showed the increase in membrane deflection with increasing applied pressure (Fig. 2c). Importantly, the PDMS membrane contacts the bottom of the flow channel at 30, 25 and 20 psi for devices with PDMS spin-coating speeds of 1000, 1200 and 1600 rpm respectively. By quantifying the deflection of the membrane, we found that the deflection percentage is linear with the applied pressure for a given PDMS membrane thickness (Fig. 2d). When the PDMS membrane thickness is reduced by increasing spin-coating speed, we found a larger deflection at the same applied pressure. Both of these results agree with a mechanical intuition of plate deflection.

To identify the ideal spinning speed and membrane thickness for the microfluidic device, we considered two criteria, which were the strength of PDMS bonding and the strength of the membrane from rupturing. Although the PDMS substrates were oxygen-plasma treated before being bonded together, a high enough air pressure could break the PDMS bonding, leading to a blockage of the microfluidic channel. Therefore, a thinner membrane was preferable under this consideration because thinner PDMS membrane completely blocked the microfluidic channel at a lower applied pressure. However, when the PDMS membrane was too thin, the device fabrication process became more challenging due to bonding issues. Based on these considerations, we fabricated and worked with microfluidic devices with a 1200 rpm PDMS spinning speed on the flow layer silicon mold, from which the membrane deflects and completely blocks the flow channel at 25 psi.

### Trapping of double emulsions

**Figure 4:**
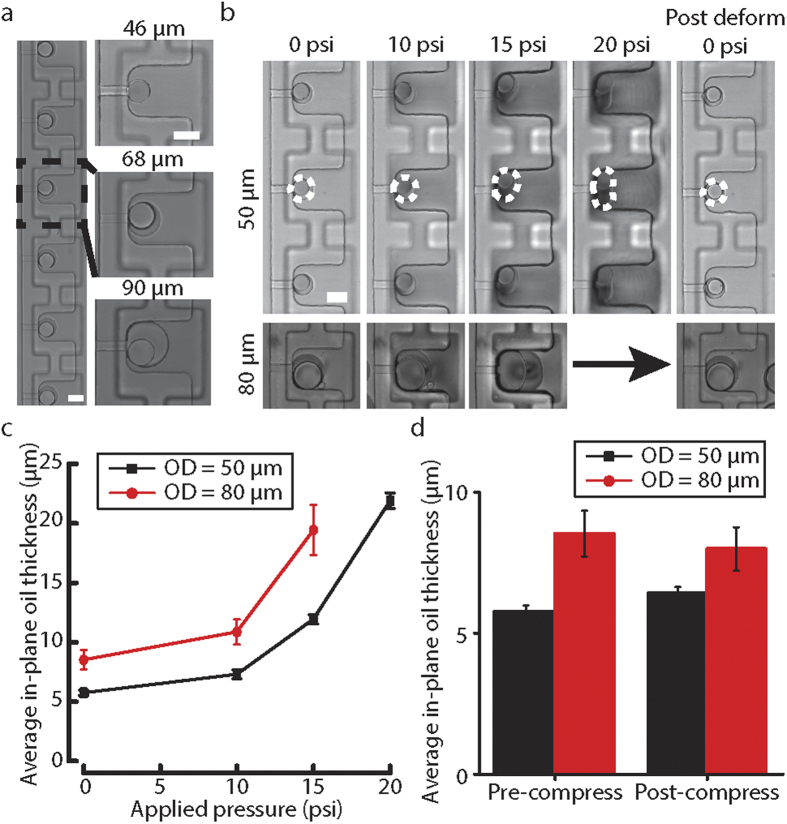
Trapping and compression of double emulsions inside trapping chambers. **(a)** Double emulsions of different sizes were trapped in the trapping chambers, scale bar = 50 μm. **(b)** Double emulsions trapped inside trapping chambers were deformed at different applied air pressures on Control valve set 2. The double emulsion droplets were squashed upon compression and returned back to spherical shape after compression. **(c)** The changes in average in-plane thickness of the middle oil phase as a function of applied pressure for two different sized droplets (n = 3, mean ± SEM). Average in-plane thickness is calculated by averaging the largest thickness and the smallest thickness of the double emulsion. **(d)** The average in-plane thickness of the middle oil phase for two different sized droplets before and after compression (n = 3, mean ± SEM).

Double emulsions were trapped inside the microfluidic device for the application of mechanical forces. Double emulsions with a diameter between 40 μm and 90 μm were first generated in a glass capillary microfluidic device (Fig. S1) and then reinjected into the microfluidic device at 10x dilution. To trap double emulsions in the trapping chambers, Control valve set 1 was then pressurized at 10–15 psi to block the main microfluidic channel, which increased the flow resistance in the main microfluidic channel. As a result, the flow resistance ratio between the microchannels and the main microfluidic channel will reduce and more fluid streamlines will go through the microchannels. This greatly increases the trapping efficiency of the double emulsions and we typically find all 14 trapping chambers filled up within a minute. A double emulsion will be trapped if the radius of the double emulsion is smaller than the instantaneous critical stream width, which is dictated by the flow resistance ratio between the micropipette and main microfluidic channel^40^. Therefore, a double emulsion with larger diameter is usually more difficult to trap; however, with actuation of Control valve set 1, the microfluidic device successfully trapped double emulsions of 46, 68 and 90 μm in diameter, as shown in Fig. 4a.

### Reversible compression of double emulsions

After demonstrating the successful trapping of double emulsions, we next evaluated the effect of compression on double emulsions. Control valve set 2 was designed to compress double emulsions inside the trapping chambers. Due to the fluid properties of the inner aqueous and middle oil phases, double emulsions deform easily when they experience different external flow fields^41^,^42^. But when the external load is removed, double emulsions return to their original spherical shapes due to interfacial tension. When we increased the applied pressure in Control valve set 2 and compressed the trapped double emulsions, the middle oil phase in-plane thickness increased. Double emulsions of 50 and 80 μm in diameter were compressed using an air pressure up to 20 and 15 psi respectively (Fig. 4b). At 20 psi air pressure, the 80 μm double emulsions escaped from the trapping chambers due to the confined space inside the trapping chambers and their larger sizes. For both sized double emulsions, compression changed their shape and oil thickness. Figure 4c shows the increase in average in-plane thickness of the middle oil phase with increasing applied pressure. The double emulsions with outer diameter of 50 μm had a 3.8 fold increase in middle phase thickness, while a 2.3 fold increase was observed for 80 μm double emulsions. The change in average in-plane thickness of the middle oil phase was completely reversible when pressure was reduced back to 0 psi after compression (Fig. 4d). Together these results showed the capability of the microfluidic device to compress double emulsions to increase their middle oil phase thickness.

### Oil removal by aspiration of double emulsions

**Figure 5:**
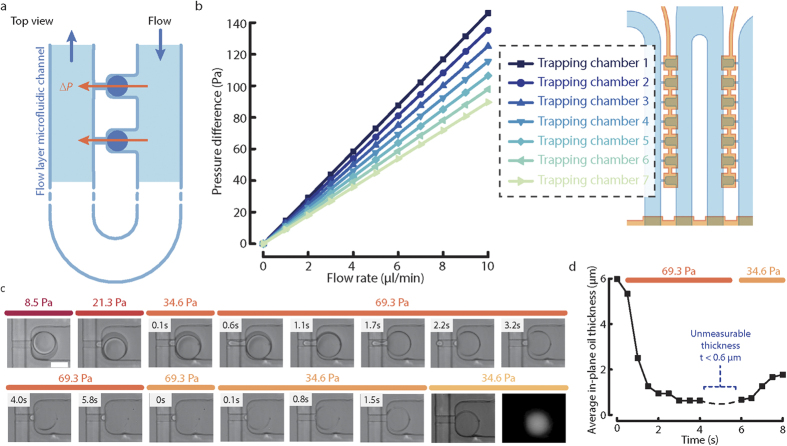
Thinning of the middle phase in double emulsion droplets by aspiration. **(a)** The flow layer microfluidic channel is designed such that trapped droplets in the trapping chambers experience a pressure difference induced by fluid flow in the microfluidic channel. **(b)** The relationship between flow rate and pressure difference at different trapping chamber position was obtained from COMSOL simulation. **(c)** A double emulsion droplet trapped in the trapping chamber was aspirated by changing the pressure difference. As the pressure difference increased, the oil changed from being aspirated to pinching off and thinning out. When the pressure difference was reduced, the oil retracted. After the thinning of oil, the double emulsion remained inside the trapping cup and the initially encapsulated fluorescent dye remained in the double emulsion. **(d)** The change in average in-plane thickness of the middle oil phase with respect to time for the same double emulsion shown in (**c**) at a pressure difference of 69.3 and 34.3 Pa.

Double emulsions are very stable and oil must be removed in order to thin out the oil phase. We postulated that aspiration of the middle oil phase could result in permanent thinning of the oil phase. Aspiration is achieved with the same mechanism used in μFPA device previously^34^. The pressure difference across the microchannel comes from the pressure drop through the main microfluidic channel due to fluid flow (Fig. 5a), which is the product of the volume flow rate and flow resistance of the main microfluidic channel. To determine the relationship between flow rate and pressure difference for each trapping chamber for our relatively complex microfluidic channel design, we performed flow simulation of the flow channel using COMSOL. As shown in Fig. 5b, the pressure difference is linear with the flow rate for each trapping chamber, with the largest pressure difference for the most upstream trapping chamber.

To test the effect of aspiration on double emulsions, we encapsulated rhodamine succinimidyl dye in 80 μm double emulsions that had an average in-plane oil thickness of 8.2 μm. By increasing the flow rate, we aspirated on double emulsions and observed several behaviors through dynamic changes of aspiration pressure, as shown in Fig. 5c. At low aspiration pressures of 8.5 or 21.3 Pa, oil was aspirated into the micropipette. Interestingly, when the pressure difference increased from 34.6 to 69.3 Pa, oil began to pinch off and the middle phase thinned out over several seconds. The transition of oil aspiration to pinching off occurs when the protrusion length of the double emulsion is larger than the hydraulic radius of the aspiration micropipette. After applying a high pressure difference to the double emulsions for several seconds (69.3 Pa, >5.8 s), the oil in the double emulsion became nearly invisible by brightfield imaging. However, there is some residual oil remained inside the micropipette channel. When the pressure difference was reduced back to 34.6 Pa, the oil retracted from the micropipette channel and refilled the double emulsion middle phase (34.6 Pa, 1.5 s). The double emulsion remained stably trapped after thinning of the oil phase, as evident by the intact encapsulated fluorescent dye in the aqueous inner phase. The average in-plane thickness of the middle oil phase of this single double emulsion was measured at aspiration pressure differences of 69.3 and 34.6 Pa (Fig. 5d). This experiment demonstrated that the middle oil phase can be thinned out permanently by aspiration in the microfluidic device.

### Mechanically activated double emulsion through aspiration and osmotic shock facilitates calcium transport through oil

Through compression and aspiration, the microfluidic device enables transient thickening and permanent thinning of the middle oil phase in double emulsions. The middle oil phase in a double emulsion behaves like a semi-permeable membrane through which solute molecules can diffuse into and out of the inner aqueous phase. This is especially relevant in drug delivery application, where non-ionized hydrophobic drug diffuses through the oil obeying Fick’s law of diffusion. Since ions are hydrophilic, they cannot diffuse through oil easily. However, in the presence of an osmotic pressure difference during hypo-osmotic shock, it has been observed that ions can also transport along with water through Span80-stabilized oil phase of double emulsions via two different mechanisms^43^. When there is no contact between the two interfaces, reverse micelles form in the presence of lipophilic surfactant molecules and diffuse through the oil, while the transport of water is carried by single hydrated surfactant molecules when there is contact between the two interfaces^43^,^44^,^45^. The ion transport was also found to be dependent on the oil-soluble surfactant^43^. Fluorinated oil (HFE-7500) with fluorosurfactants (PFPE-PEG) used to generate double emulsions in this study has been shown to provide permeability to oxygen and other non-ionized small molecules^46^,^47^. However, the transport of ions through this oil is not known. Thus, we investigated ion transport through the middle oil phase as a response that involves mechanically perturbing double emulsions.

**Figure 6:**
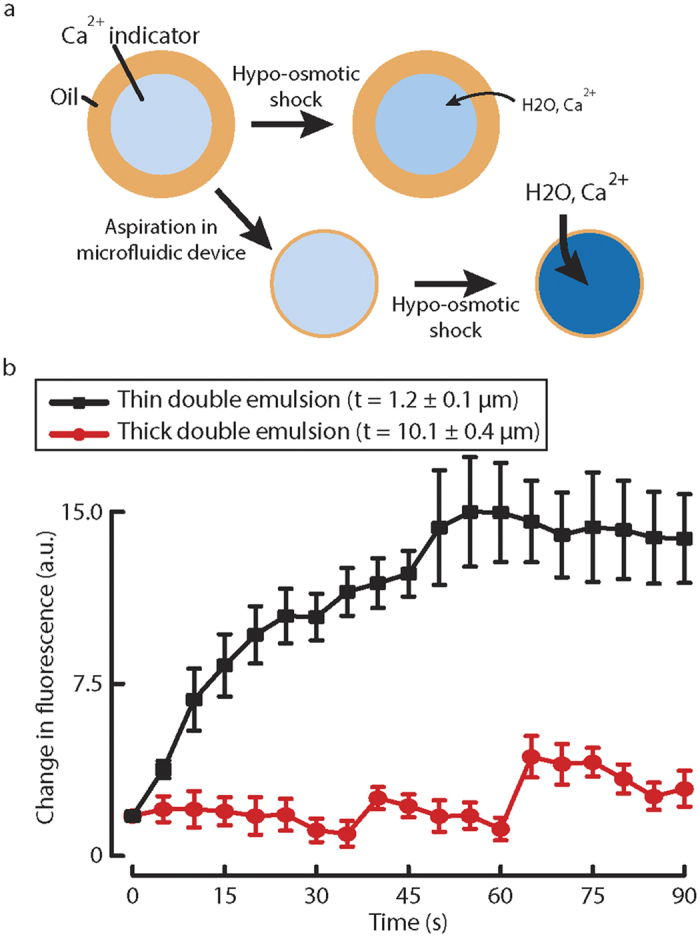
Mechanically activated artificial cell through aspiration and osmotic shock facilitates calcium ion transport through oil. (**a**) Thick double emulsions are aspirated in the microfluidic device to form thinner double emulsions. Both thick and thin double emulsions are hypo-osmotically shocked, such that calcium ions diffuse through the oil and enter the double emulsions. (**b**) The changes in fluorescence as a function of time for thick and thin double emulsions (n = 12, mean ± SEM).

In order to detect transport of calcium ions during osmotic downshock, we encapsulated Rhod-2 calcium indicator in double emulsions (Fig. 6a). We did not find a significant change in fluorescence when we subject thick double emulsions with hypo-osmotic shock (Fig. S2). However, when we aspirated the double emulsions to permanently thin out the oil, calcium ions entered the double emulsions rapidly as indicated by the increase in fluorescence for the calcium indicator (Fig. 6b). By comparison, unaspirated double emulsions that had a thicker middle phase did not have increased fluorescence during osmotic downshock over the same time period. This indicates that by aspirating double emulsions using the microfluidic device, calcium ions in the outer phase entered the double emulsions faster. The transport of ions through the fluorinated oil of this emulsion system behaved differently when compared to that through hexadecane with Span80^43^. Nevertheless, we demonstrated influx of calcium ions by mechanical activation of double emulsion artificial cell.

## Conclusion

We have successfully demonstrated mechanically activated artificial cell using a newly designed microfluidic device. The prototype of artificial cell is a double emulsion with oil separating the inner and outer aqueous phases. We showed that we can control oil thickness by compression and aspiration as a way to mechanically regulate transport process in artificial cells. This represents the first demonstration to couple a mechanical input to an output in double emulsion as an artificial cell. We envision testing the possibility of mechanically regulating gene expression in artificial cells encapsulating cell-free expression system. Our work also highlights the power and utility of microfluidic devices in synthetic biology research. Although our mechanosensitive artificial cell is quite primitive at this point, it is a starting point for further engineering. All life forms are mechanosensitive, as mechanical force influences biochemistry^48^, and our work opens up exciting opportunities in force-activated synthetic biology.

## Methods

### Glass capillary microfluidic device

Glass capillary microfluidic device was fabricated by assembling tapered round capillary, square capillary and syringe needles together using 5 minutes epoxy (Fig. S1)^49^. First, the round glass capillary (Sutter Instrument B100-58-10) was pulled using a pipette puller (Sutter Instruments P-87). The tapered capillary was then sanded using 1200 grade sandpaper to obtain a 100 μm opening for the collection tube. The outside surface of a clean 15 μm opening injection tube, pulled and similarly sanded, was treated with trichloro(1H,1H,2H,2H-perfluorooctyl)silane to render the surface hydrophobic. Then, the injection tube and collection tube were inserted into a square capillary (AIT, 810-9917) placed on a glass slide and they were aligned under an optical microscope. Syringe needles (McMaster Carr, 75165A677) were cut and glued to the glass slide using 5 minutes epoxy (Devcon, 14250). Lastly, micro-tubings (Scientific Commodities, BB31695-PE/5) were connected to the syringe needles.

### Double emulsions generation

For a stable double emulsion system, we used fluorinated oil (HFE-7500, RAN Biotechnologies) with fluorosurfactants, which was synthesized by coupling oligomeric perfluorinated polyethers (PFPE) with polyethyleneglycol (PEG)^50^, as the middle phase. Monodisperse double emulsions were generated using the glass capillary microfluidic device described in the previous section. The inner aqueous phase contained 20 μM rhodamine succinimidyl dye (Fisher scientific, 50-851-056) or 100 μM Rhod-2 (Thermo Fisher Scientific, R-14220), 800 mM glucose, 20 mM HEPES pH7.5, 250 μM EGTA. The middle oil phase contained 2 wt% 008-FluoroSurfactant in HFE7500. The outer aqueous phase contained 10 wt% Poly(vinyl alcohol) (PVA) (Sigma, MW 13,000–23,000, 87–89% hydrolyzed) or 8 wt% PVA, 300 mM CaCl_2_, 20 mM HEPES pH7.5, 250 μM EGTA. The three phases were pumped into the glass capillary microfluidic device using three OEM syringe pumps (New Era Pump Systems, NE-500). For the 65 μm double emulsion that had an oil thickness of 11 μm, the flow rate used for the inner, middle and outer phases were 450, 700, 6000 μl/hr respectively. The flow rate of the middle phase and outer phase were set at 400 and 8000 μl/hr for a thinner and smaller double emulsion; and at 900 and 5000 μl/hr for a thicker and larger double emulsion.

### Device fabrication

The microfluidic device was fabricated using multilayer polydimethylsiloxane (PDMS) soft-lithography technique^51^. The microfluidic device is composed of a PDMS control layer and a PDMS flow layer, which were aligned and bonded permanently onto a PDMS coated microscope glass slide. Three photomasks were produced by high resolution inkjet printing on transparency film (CAD/Art Services) for SU-8 patterning of two silicon molds by standard photolithography. The SU-8 patterning procedures of the two silicon molds followed the standard protocol developed by Microchem, Inc. for SU-8 2000. After the silicon molds were fabricated, they were casted with PDMS (Sylgard-184). The control layer was casted in a mixing ratio of 7:1 (base:curing agent). The flow channel membrane was generated by spin-coating PDMS with a mixing ratio of 20:1 on the flow layer silicon mold. After the PDMS control substrate was diced and punched holes, both the diced PDMS control substrate and the PDMS flow layer membrane on the silicon mold were placed in an oxygen plasma etcher. The diced PDMS control layer substrate was then carefully aligned and bonded with the PDMS flow layer membrane using a customized alignment platform on an optical microscope. The day after, the bonded control layer substrate with the flow layer membrane was cut out. Holes were punched at the inlets and outlets. Finally, the PDMS substrate was bonded to a PDMS-coated microscope glass slide. Schematic of the fabrication process flow of the microfluidic device can be found in Fig. S3 and more detailed fabrication steps can be found in Supplementary Method.

### Imaging membrane deflection and 3D image reconstruction

The microfluidic device was mounted on a spinning disk confocal microscope (Olympus IX73 with Yokogawa CSU-X1) for image acquisition using a 20X objective. Four different micro-tubings were connected to the microfluidic device. The control layer inlets of the microfluidic device were connected through 4-way stopcock switches (Nordson, FC series) to a compressed air source with a pressure regulator (Norgren). Rhodamine succinimidyl dye (Fisher scientific, 50-851-056) was flowed into the device through the inlet of the flow channel using a syringe pump. By setting the position of the stopcock switches, compressed air of pressure ranging from 0 psi to 30 psi was applied to deflect the PDMS membrane above the trapping chambers. A z-series of fluorescent images, excited at 561 nm, was captured at a step size of 1 μm and was reconstructed in ImageJ to generate 3D and side view images.

### Imaging double emulsions trapping, compression and aspiration

The microfluidic device was mounted on an optical fluorescent microscope (Nikon, Ti Eclipse) for image acquisition using a 10X objective. The control layer inlets were connected to a compressed air source. Double emulsions were first formed as described in the glass capillary microfluidic device, encapsulating 20 μM rhodamine succinimidyl dye, and then flowed into the microfluidic device using a syringe pump at 10X dilution at 10 μl/min. For trapping double emulsions, once they reached the flow channel, the flow rate was reduced to 0.2–0.5 μl/min and 10–15 psi pressure was applied to Control valve set 1, which blocks the flow through the main microfluidic channel. Control valve set 1 was decompressed after the double emulsions were trapped in the trapping chambers, typically in under a minute. For double emulsion compression, the stopcock switches were then configured to apply compressed air to Control valve set 2. The applied air pressure varied from 0 to 30 psi. For double emulsion aspiration, no air pressure was applied to neither of the control valve sets. The pressure difference across the double emulsions were controlled by varying the flow rate in the flow channel.

### Flow simulation

The fluid flow in the flow channel of the microfluidic device was simulated using COMSOL 4.4 (COMSOL Multiphysics). The three dimensional model of the device was constructed in Solidworks with the height of the micropipettes and main microfluidic channel as measured from the flow channel SU-8 pattern. The pressure differences across trapping chambers were simulated using the laminar flow module. The problem is computed as incompressible flow. Water was chosen as the fluid material of the entire domain. No-slip boundary conditions were used on all the walls except the inlet and outlet. The inlet was set as normal inflow velocity, which was calculated from the flow rates and cross-sectional area of the inlet. A zero constant pressure with backflow suppression was used at the outlet.

### Hypo-osmotic shock experiment for calcium transport

Double emulsions were generated as described in the glass capillary microfluidic device, with 100 μM calcium indicator Rhod-2, 800 mM glucose, 20 mM HEPES pH7.5, 250 μM EGTA as the inner phase and 8 wt% PVA, 300 mM CaCl_2_, 20 mM HEPES pH7.5, 250 μM EGTA as the outer phase. The osmolarity of the inner and outer phase were measured using a Vapor Pressure Osmometer (ELITechGroup, 5600) and were balanced. The microfluidic device was mounted on an optical fluorescent microscope with a 10X objective and the control layer was connected to the pressure regulator as described before. The flow channel inlet was connected to two syringe pumps using a T-junction connector. Two syringes with double emulsion solution and a hypo-osmotic solution (8 wt% PVA, 200 mM CaCl_2_, 20 mM HEPES pH7.5, 250 μM EGTA) were connected to the two syringe pumps respectively. First, the double emulsions were pumped into the device and trapped inside the trapping chambers. Then, the outer phase was changed to the hypo-osmotic solution. The trapped double emulsions were aspirated and thinned out by increasing the flow rate of the hypo-osmotic solution. The fluorescence of the inner phase was monitored at a time interval of 5 s, and images were analyzed in ImageJ.

## Additional Information

**How to cite this article**: Ho, K. K. Y. *et al*. Mechanically activated artificial cell by using microfluidics. *Sci. Rep*. **6**, 32912; doi: 10.1038/srep32912 (2016).

## Supplementary Material

Supplementary Information
