# Supplementary material for: Mechanically activated artificial cell by using microfluidics
Source: Sci Rep. 2016 Sep 9;6:32912. doi: 10.1038/srep32912 (PMC5017192; doi:10.1038/srep32912)
Supplement: Supplementary Information [file srep32912-s1.pdf]

Mechanically activated artificial cell by using microfluidics

Kenneth K.Y. Ho, Lap Man Lee, Allen P. Liu

Supplementary method

Detailed device fabrication

Three photomasks were produced by high resolution inkjet printing on transparency film (CAD/Art Services) for SU-8 patterning of two silicon molds by standard photolithography. The first silicon mold is composed of one layer of SU-8 pattern for PDMS casting of the control layer. The SU-8 pattern of the control layer defined two sets of integrated microfluidic control valves for closing of the main microfluidic channel (Control valve set 1) and deflections of the flow layer in the trapping chambers for planer compression of double emulsions (Control valve set 2) respectively. These two sets of microfluidic control valves contained two independent inlets. The other silicon mold is composed of two layers of SU-8 patterns to be used for PDMS spin-coating of the flow layer. The first SU-8 patterning layer defined the side microfluidic pipette channel for aspiration. The second SU-8 patterning defined the main microfluidic channel, the trapping chambers, and a single pair of inlet and outlet.

First, two silicon wafers were dehydrated on a hotplate at 200 ^{o}C for 15 min to promote photoresist adhesion. In SU-8 patterning the silicon mold for the control layer, SU-8 2025 was spin-coated at 1500 rpm onto the wafer for 30 s, which gave a thickness of 45 μm. The photoresist was then exposed to UV light for 20 s under a contact aligner (Karl Suss, MJB45). In the first SU-8 patterning of the flow layer, SU-8 2010 was spin-coated at 1500 rpm onto the silicon wafer for 30 s, which gave a thickness of 16 μm. The photoresist was then exposed to UV light for 15 s with the first flow channel photomask, which defined the side pipette arrays. After development of first SU-8 layer, the silicon wafer was hard-baked at 200 ^{o}C for 20-30 minutes to consolidate the developed SU-8 patterns before the application of the second SU-8 layer. Subsequently, for the second layer SU-8 patterning of the flow layer, SU-8 2025 was spin-coated at 1500 rpm onto the silicon wafer for 30 s, which gave a combined thickness of 60 μm. The photoresist was then aligned with the second flow channel photomask, which mainly defined the main microfluidic channel, and exposed to UV light for 20 s. After development of SU-8, both silicon molds were hard-baked at 200 ^{o}C for 20-30 minutes again to cure any surface cracks. The thicknesses of both the control layer and flow channel SU-8 pattern were measured using a stylus profilometer (Dektak 6M).

Before PDMS casting or spin-coating on the silicon molds, both wafers were first silanized with trichloro(1H,1H,2H,2H-perfluorooctyl)silane (Sigma-Aldrich) in a desiccator for 1 hour. The silicon mold for the control layer was casted with PDMS (Sylgard-184) with a mixing ratio of 7:1 (base:curing agent). After degassing in a desiccator, the control layer PDMS substrate was then cured at 60 ^{o}C overnight before demolding from the wafer. The PDMS substrate was then diced and holes were punched with 1 mm diameter at the inlets of the microfluidic control valves. The flow channel membrane was generated by spin-coating PDMS with a mixing ratio of 20:1 (base:curing agent) on the flow layer silicon mold at rotational speeds between 1000 to 1600 rpm for 30 s. After this, the PDMS flow layer membrane was cured at 60 ^{o}C for 2 hours. Both the diced PDMS control substrate and the PDMS flow layer membrane on the silicon mold were placed in an oxygen plasma etcher (Femto, Covance) to render the PDMS surfaces hydrophilic for the preparation of bonding procedure described as follows. The flow layer silicon mold containing the PDMS membrane was mounted on a customized alignment platform on an optical microscope. The diced PDMS control layer substrate was then careful aligned and bonded with the PDMS flow layer membrane. The permanent bonding between the control layer substrate and PDMS flow layer membrane was established by heating in the furnace at 60 ^{o}C overnight with the aid of gentle pressing between two substrates. The day after, the bonded control layer substrate with the flow layer membrane was then cut out and peeled off from the flow layer silicon wafer. Inlet and outlet holes (1 mm diameter) for the main microfluidic flow channel were punched through the layer PDMS control/flow substrate. Finally, the PDMS substrate was bonded to a PDMS-coated microscope glass slide following the procedures of two-hour PDMS curing and oxygen plasma treatment as described previously. Schematic of the fabrication process flow of the microfluidic device can be found in Figure S3.

Supplementary figure 1

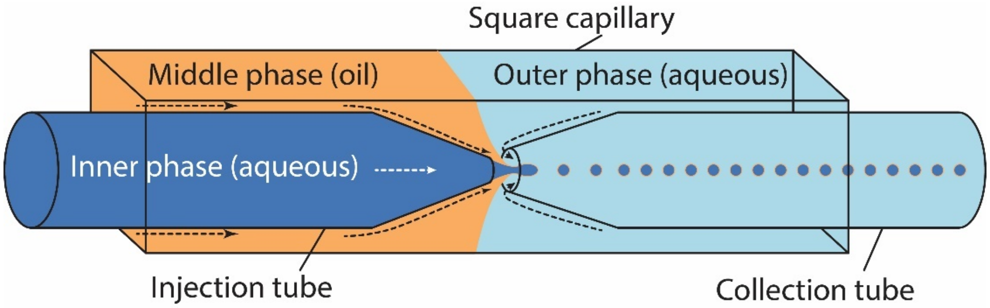

Figure S1: Glass capillary microfluidic device for formation of double emulsions. The glass capillary microfluidic device is fabricated by aligning two tapered round capillaries inside a square capillary. Monodisperse double emulsion droplets form when the inner aqueous phase, middle oil phase and outer aqueous phase are pumped into the device in the corresponding compartments.

Supplementary figure 2

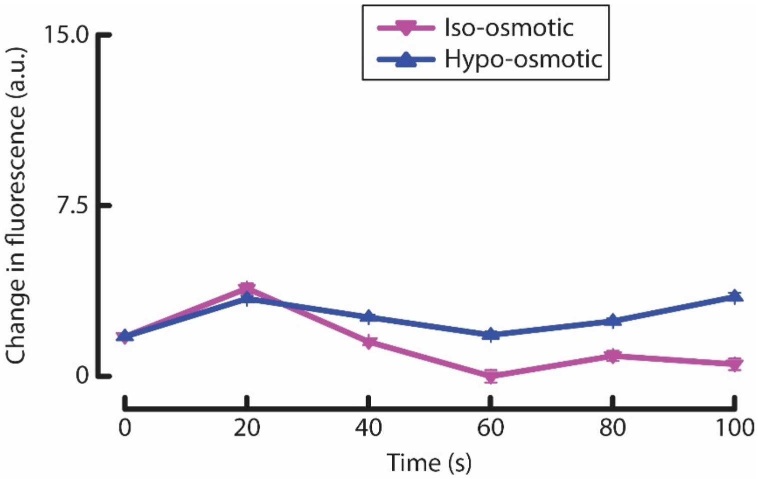

Figure S2: Transport of calcium ions across the oil phase in thick double emulsions. The changes in fluorescence level as a function of time for thick double emulsions (Thickness = mean ± S.E.: t = 10.7 ± 0.1 μm) in iso- and hypo-osmotic solution. (n = 10)

Supplementary figure 3

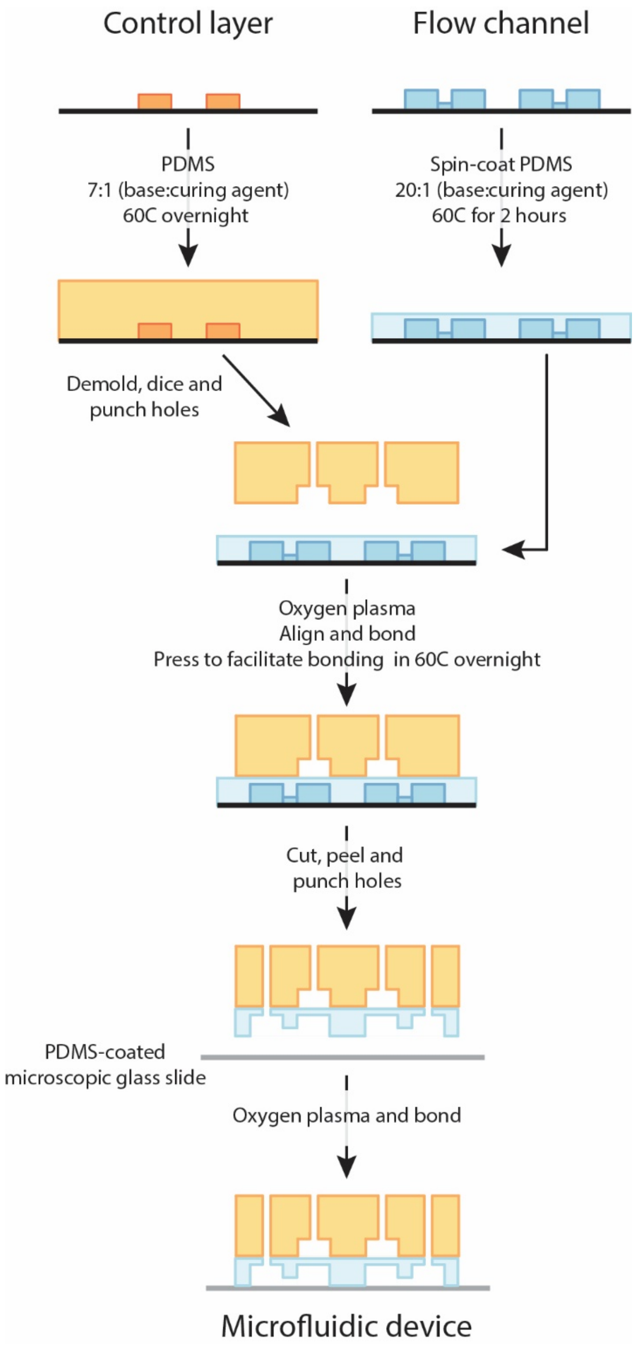

Figure S3: Fabrication process of microfluidic device. The silicon mold for the control layer was casted with PDMS with a mixing ratio of 7:1 (base:curing agent). After curing at 60 °C overnight, the control layer PDMS substrate was demolded, diced and punched holes at the inlets of the microfluidic control valves. The flow channel membrane was generated by spin-coating PDMS with PDMS with a mixing ratio of 20:1. After curing at 60 °C for 2 hours, the diced PDMS control layer substrate was aligned and bonded with the PDMS flow layer membrane. Gentle pressing between two substrates was applied in the oven at 60 °C overnight. Then, the PDMS control/flow substrate was cut, peeled and punched holes at the main microfluidic channel inlet and outlet. Finally, the PDMS substrate was bonded to a PDMS-coated microscope glass slide.
